# Controlled diabetes amends oxidative stress as mechanism related to severity of diabetic retinopathy

**DOI:** 10.1038/s41598-021-96891-7

**Published:** 2021-09-03

**Authors:** Rania Fahmy, Nouf M. Almutairi, May N. Al-Muammar, Ramesa Shafi Bhat, Nadine Moubayed, Afaf El-Ansary

**Affiliations:** 1 Department of Optometry, College of Applied Medical Sciences, King Saud University, Riyadh, Saudi Arabia; 2 Department of Ophthalmology, Faculty of Medicine, Cairo University, Giza, Egypt; 3 Department of Optometry, King Saud Medical City, Riyadh, Saudi Arabia; 4 Clinical Nutrition Department, College of Applied Medical Sciences, King Saud University, Riyadh, Saudi Arabia; 5 Biochemistry Department, College of Science, King Saud University, Riyadh, Saudi Arabia; 6 Botany Department, College of Science, King Saud University, Riyadh, Saudi Arabia; 7 Central Laboratory, Female Center for Medical Studies and Scientific Section, King Saud University, Riyadh, Saudi Arabia; 8 Therapeutic Chemistry Department, National Research Centre, Dokki, Cairo, Egypt

**Keywords:** Biochemistry, Biomarkers, Diseases, Health care

## Abstract

Oxidative stress is a well-accepted etiological mechanism that contributes to neuronal dysfunction. Role of oxidative stress as a mechanism of retinopathy in controlled type 2 diabetic patients was evaluated. Participants were divided into three groups: Group 1 as 30 normal eyes of 15 subjects, Group 2 comprised 24 eyes of 12 diabetic patients without retinopathy and Group 3 comprised 23 eyes of 12 diabetic patients with different grades of retinopathy (8 eyes with maculopathy). A complete ophthalmological examination was performed. Oxidative stress markers were measured in blood. Macular thickness was different in all quadrants among all groups and showed a tendency to increase in Group 3 due to diabetic retinopathy with insignificant changes in parapapillary retinal nerve fiber layer thickness although thinning was noted also with retinopathy. Non-significant differences in GST and lipid peroxide levels were observed between the three studied groups, whereas vitamin C and GSH levels were higher in diabetic patients when compared to those in controls. As oxidative stress, hyperglycemia and local inflammation are involved in the pathogenesis of DR, the present study proved that the progressive damage can be retarded in controlled type 2 diabetic patients using different treatment modalities that abated oxidative stress.

## Introduction

Diabetes mellitus is a group of metabolic diseases characterized by hyperglycemia due to defects in insulin production and/or insulin action together with impaired carbohydrates, lipids, and protein metabolism. It has long term health complications due to the damage, dysfunction, and failure of different organs^1^. In addition to the kidneys, blood vessels, nerves, and heart, the eyes are greatly affected leading to diabetic retinopathy. However, diabetic patients vary in their predisposition to the development of these complications^2^. The appearance, timing, and severity of each complication in a given patient are largely determined by these factors. Retinopathy is considered to be a predictive status of cardiovascular disease (CVD), and demonstrates fivefold higher risk of developing CV complications^3^. The genetic hypothesis suggests that complications from diabetes are genetically controlled; the metabolic hypothesis suggests that complications such as cellular and vascular damage are mostly relative to the level and duration of hyperglycemia^4^. Ethnicity influences the rate of development of diabetes-related complications. Complications can be delayed and reduced by maintaining tight glycemic control, avoiding oxidative stress, and local inflammation^2^.

It is still not clear why these complications develop in some patients with poor glycemic control and not in others. Previous studies suggested that hyperglycemia-induced cellular damage, oxidative stress and reactive oxygen species (ROS) are key players for mediating the development of diabetic complications, including retinopathy^5^. Several factors including, ethnicity, genetic susceptibility to damage, environmental factors and co-morbidities are contributors to the predisposition to DM complications. ROS cause strand breaks in nuclear DNA and activate downstream pathways that lead to pancreatic β cell damage. This mechanism influences microvascular and macrovascular complications^6,7^.

Previous studies have demonstrated that in T2DM, hyperglycemia-induced polyol pathway flux stimulates ROS interaction within the cell^8,9^. The changes in the polyol pathway flux include increased aldose reductase enzyme activity thus resulting in excessive sorbitol and NADP + ^10^. The changed metabolic environment usually leads to a decrease in cellular NADPH thereby leading to a reduction in NADPH dependent synthesis of the protective antioxidant glutathione (GSH)^11,12^.

Based on the fact that several oxidative stress biomarkers have been identified to play a critical role in the pathogenesis of T2DM, and that many of these markers affect retinopathy as a curve status of CVD, the objective of this study is to find the contribution of GSH, vitamin C, GST together with lipid peroxides as markers of antioxidant/oxidative stress signaling in the development of retinopathy in medically controlled T2DM.

## Results

A total of 77 eligible eyes of 39 participants were enrolled into the study. One eye in Group 3 was not analyzed because of media opacity. Table 1 lists the demographic data from 15 control participants (Group 1) and 24 diabetic participants 12 without and 12 with retinopathy (Group 2 and 3, respectively). Age, intraocular pressure, and refractive error that affect the RNFLT were not statistically different among the groups.

**Table 1:** Demographic features of control and controlled diabetic patients with and without retinopathy.

Variables	Group 1 (mean ± SD)	Group 2 (mean ± SD)	Group 3 (mean ± SD)
Age (years)	47.2 ± 4.69 (40–58)	46.83 ± 6.14 (40–56)	52.41 ± 6.34 (40–60)
Duration of DM (years)		7.5 ± 8.14	12.58 ± 8.08
IOP (mmHg)	17.86 ± 3.62	18.5 ± 2.85	20.20 ± 2.65
Visual acuity (log Mar)	0.08 ± 0.21	0.06 ± 0.12	0.24 ± 0.34
Refractive error (diopters)	−1.08 ± 2.84	−0.61 ± 1.31	−4.97 ± 15.48

The inner and outer macular thicknesses in four quadrants around the macula in both eyes (OD &OS) were measured by 3D- OCT 2000 is shown in Tables 2 and 3. The macular thickness was different in all quadrants among all groups and tends to increase in Group 3 due to DR except in inner superior quadrants of both eyes. However, a comparison of the macular thickness in the four quadrants among all groups (b P) revealed no statistical difference except in the inner and outer inferior and outer temporal quadrants of the right eye (P = 0.085, 0.083 and 0.031 respectively) and the inner inferior quadrant of left eye (P = 0.044). Only the inner inferior macular thickness of the left eye was significantly different in Group 2 compared to Group 1 (a P = 0.049).

**Table 2:** Outer and inner macular thickness (µm) in four quadrants of right eye (OD) of controlled diabetic patients with and without retinopathy, compared to healthy control subjects.

Parameter		Group	N	Min	Max	Mean ± SD	% Change	P value^a^	P value^b^
I	Out	Group I	15	231.00	294.00	264.67 ± 14.39	100.00		0.085
		Group II	12	222.00	297.00	262.25 ± 22.99	99.09	0.976	
		Group III	11	244.00	445.00	291.27 ± 55.73	110.05	0.100	
	In	Group I	15	286.00	322.00	299.80 ± 9.76	100.00		0.083
		Group II	12	233.00	332.00	278.42 ± 27.94	92.87	0.221	
		Group III	11	261.00	469.00	311.73 ± 57.87	103.98	0.614	
S	Out	Group I	15	235.00	284.00	268.00 ± 12.96	100.00		0.274
		Group II	12	232.00	296.00	258.42 ± 17.48	96.42	0.316	
		Group III	11	236.00	308.00	269.82 ± 24.54	100.68	0.957	
	In	Group I	15	257.00	321.00	302.80 ± 14.96	100.00		0.209
		Group II	12	250.00	342.00	287.92 ± 23.99	95.08	0.219	
		Group III	11	230.00	332.00	288.36 ± 33.83	95.23	0.253	
N	Out	Group I	15	252.00	301.00	280.27 ± 13.83	100.00		0.157
		Group II	12	200.00	308.00	268.92 ± 27.28	95.95	0.264	
		Group III	11	261.00	323.00	284.73 ± 17.85	101.59	0.805	
	In	Group I	15	291.00	319.00	305.93 ± 9.60	100.00		0.241
		Group II	12	248.00	340.00	292.75 ± 23.98	95.69	0.183	
		Group III	11	264.00	345.00	297.18 ± 26.16	97.14	0.464	
T	Out	Control	15	242.00	272.00	260.87 ± 9.13	100.00		0.031
		Group I	12	218.00	289.00	248.75 ± 18.18	95.36	0.405	
		Group II	11	245.00	390.00	279.27 ± 44.37	107.06	0.158	
	In	Group I	15	273.00	387.00	295.60 ± 27.01	100.00		0.129
		Group II	12	225.00	319.00	272.08 ± 26.74	92.04	0.211	
		Group III	11	247.00	465.00	303.45 ± 57.19	102.66	0.830	
C		Group I	15	190.00	273.00	229.67 ± 20.32	100.00		0.395
		Group II	12	175.00	308.00	233.75 ± 38.49	101.78	0.955	
		Group III	11	149.00	364.00	251.55 ± 61.39	109.53	0.324	

Comparisons done using one-way ANOVA test between all groups with multiple comparisons (Dunnett test) to compare each group with the control group in all parameters.

^a^P value between each group and the control group.

^b^P value between all groups.

**Table 3:** Outer and inner macular thickness (µm) in four quadrants of left eye (OS) of controlled diabetic patients with and without retinopathy compared to healthy control subject.

Parameter		Group	N	Min	Max	Mean ± SD	%Change	P value^a^	P value^b^
I	Out	Group I	15	235.00	328.00	266.73 ± 23.70	100.00		0.972
		Group II	12	231.00	350.00	268.25 ± 33.07	100.57	0.986	
		Group III	12	230.00	324.00	269.25 ± 26.06	100.94	0.961	
	In	Group I	15	253.00	355.00	303.73 ± 23.24	100.00		0.044
		Group II	12	216.00	307.00	271.17 ± 27.35	89.28	0.049	
		Group III	12	241.00	458.00	304.58 ± 53.64	100.28	0.997	
S	Out	Group I	15	256.00	286.00	272.33 ± 9.08	100.00		0.241
		Group II	12	194.00	295.00	258.08 ± 26.57	94.77	0.253	
		Group III	12	241.00	354.00	273.50 ± 34.65	100.43	0.990	
	In	Group I	15	253.00	324.00	302.87 ± 16.51	100.00		0.413
		Group II	12	225.00	341.00	288.08 ± 30.86	95.12	0.332	
		Group III	12	253.00	366.00	299.17 ± 38.14	98.78	0.926	
N	Out	Group I	15	222.00	293.00	254.60 ± 15.83	100.00		0.478
		Group II	12	201.00	279.00	244.50 ± 22.50	96.03	0.537	
		Group III	12	218.00	374.00	257.17 ± 39.41	101.01	0.958	
	In	Group I	15	243.00	301.00	285.13 ± 16.14	100.00		0.627
		Group II	12	230.00	318.00	276.00 ± 23.98	96.80	0.657	
		Group III	12	217.00	406.00	287.00 ± 45.05	100.65	0.982	
T	Out	Group I	15	259.00	311.00	284.47 ± 13.08	100.00		0.131
		Group II	12	222.00	319.00	274.33 ± 25.02	96.44	0.473	
		Group III	12	260.00	374.00	295.08 ± 33.53	103.73	0.442	
	In	Group I	15	285.00	323.00	302.20 ± 12.90	100.00		0.412
		Group II	12	249.00	333.00	288.25 ± 26.48	95.38	0.385	
		Group III	12	254.00	409.00	302.00 ± 44.26	99.93	1.000	
C		Group I	15	186.00	364.00	236.93 ± 45.42	100.00		0.976
		Group II	12	189.00	286.00	236.50 ± 31.13	99.82	0.999	
		Group III	12	191.00	336.00	239.92 ± 46.07	101.26	0.976	

Comparisons done using one-way ANOVA test between all groups with multiple comparisons (Dunnett test) to compare each group with the control group in all parameters.

^a^P value between each group and the control group.

^b^ P value between all groups.

Table 4 demonstrates the RNFLT around the optic disc measured in both eyes (OD&OS) for all groups. Comparing the control group with diabetic groups, no significant differences were found although thinning was noted with retinopathy.

**Table 4:** Optic nerve (RNFLT) (µm) in right eye (OD); left eye (OS) of controlled diabetic patients with and without retinopathy compared to healthy control subjects.

	Parameter	Group	N	Min	Max	Mean ± SD	%Change	P value^a^	P value^b^
Right eye (OD)	I	Group I	15	231.00	294.00	264.67 ± 14.39	100.00		0.320
		Group II	12	222.00	297.00	262.25 ± 22.99	99.09	0.790	
		Group III	11	244.00	445.00	291.27 ± 55.73	110.05	0.234	
	S	Group I	15	286.00	322.00	299.80 ± 9.76	100.00		0.092
		Group II	12	233.00	332.00	278.42 ± 27.94	92.87	0.883	
		Group III	11	261.00	469.00	311.73 ± 57.87	103.98	0.139	
	N	Group I	15	235.00	284.00	268.00 ± 12.96	100.00		0.222
		Group II	12	232.00	296.00	258.42 ± 17.48	96.42	0.983	
		Group III	11	236.00	308.00	269.82 ± 24.54	100.68	0.239	
	T	Group I	15	257.00	321.00	302.80 ± 14.96	100.00		0.143
		Group II	12	250.00	342.00	287.92 ± 23.99	95.08	0.953	
		Group III	11	230.00	332.00	288.36 ± 33.83	95.23	0.113	
Left eye (OS)	I	Group I	15	104.00	170.00	134.80 ± 17.64	100.00		0.759
		Group II	12	16.00	180.00	129.75 ± 39.16	96.25	0.859	
		Group III	12	64.00	170.00	126.92 ± 25.06	94.15	0.696	
	S	Group I	15	95.00	193.00	133.80 ± 25.22	100.00		0.546
		Group II	12	62.00	153.00	129.17 ± 23.56	96.54	0.885	
		Group III	12	40.00	171.00	121.50 ± 36.52	90.81	0.449	
	N	Group I	15	46.00	95.00	70.47 ± 14.63	100.00		0.552
		Group II	12	43.00	80.00	67.50 ± 10.95	95.79	0.841	
		Group III	12	11.00	87.00	63.92 ± 19.53	90.70	0.453	
	T	Group I	15	48.00	136.00	90.20 ± 26.35	100.00		0.670
		Group II	12	59.00	125.00	89.92 ± 24.73	99.69	0.999	
		Group III	12	44.00	118.00	82.33 ± 22.82	91.28	0.636	

Comparisons done using one-way ANOVA test between all groups with multiple comparisons (Dunnett test) to compare each group with the control group in all parameters.

^a^P value between each group and the control group*.*

^b^P value between all groups.

Table 5 demonstrate the levels and the percentage changes of GSH, GST, lipid peroxides and vitamin C in controlled diabetic patients with and without retinopathy compared to healthy controls. GST and lipid peroxides did not show any significant difference between the three studied groups; vitamin C and GSH were unexpectedly higher in controlled diabetic patients compared to control participants.

**Table 5:** Mean ± SD and percentage changes of the four measured parameters in the serum of controlled diabetic patients with and without retinopathy compared to healthy control subjects.

Parameter	Group	N	Min	Max	Mean ± S.D	% Change	P value^**a**^	P value^**b**^
GST (U/ml)	Group I	15	15.10	18.75	17.00 ± 1.05	100.00		0.200
	Group II	12	13.50	18.70	16.34 ± 1.67	96.12	0.445	
	Group III	12	10.90	18.70	15.93 ± 1.86	93.72	0.143	
Lipid Peroxides (μmoles MDA/ml)	Group I	15	0.07	0.11	0.09 ± 0.01	100.00		0.128
	Group II	12	0.07	0.09	0.08 ± 0.01	94.86	0.266	
	Group III	12	0.08	0.10	0.08 ± 0.01	92.96	0.099	
Vitamin C (μg/ml)	Group I	15	10.41	21.00	15.45 ± 3.40	100.00		0.001
	Group II	12	18.00	41.00	26.58 ± 8.64	172.02	0.001	
	Group III	12	13.00	35.00	24.83 ± 7.04	160.69	0.001	
Glutathione (μg/dl)	Group I	15	63.00	93.91	75.23 ± 10.13	100.00		0.001
	Group II	12	69.00	117.00	96.51 ± 13.94	128.29	0.001	
	Group III	12	68.11	99.00	85.62 ± 10.11	113.80	0.046	

Comparisons done using one-way ANOVA test between all groups with multiple comparisons (Dunnett test) to compare each group with the control group in all parameters.

^a^P value between each group and the control group.

^b^P value between all groups.

**Figure 1:**
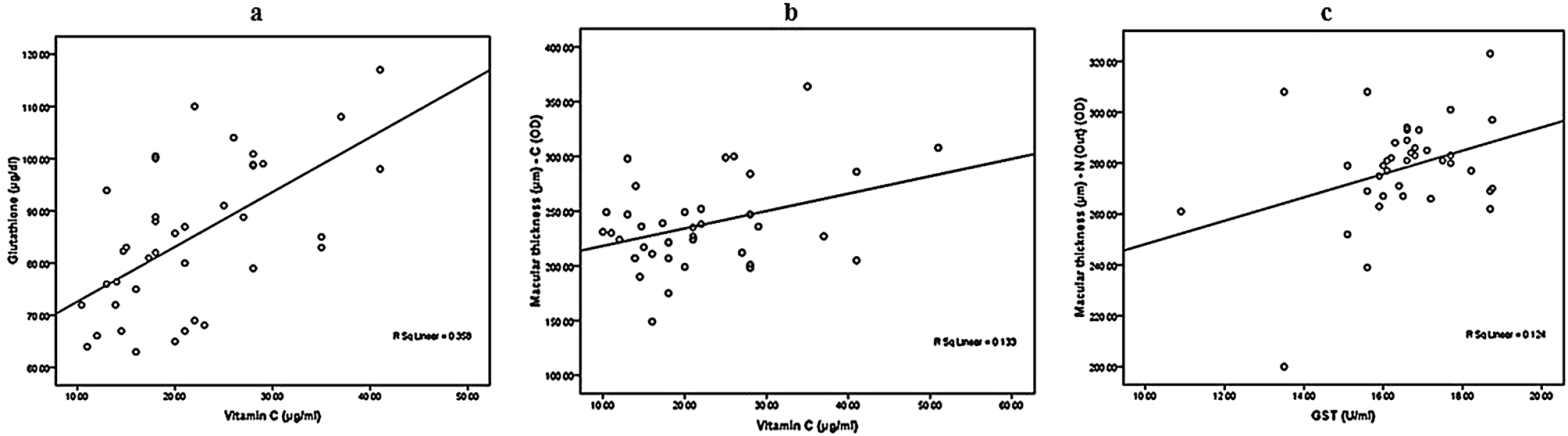
Correlation between **(a)** vitamin C (μg/ml) and glutathione (μg/dl); **(b)** vitamin C (μg/ml) and macular thickness (µm)—C (OD); **(c)** GST (U/ml) and macular thickness (µm)—N (Out) (OD) with best fit line curve (positive correlation).

Table 6 and Fig. 1 demonstrate the positive correlations between vitamin C and GST and macular thickness based on biochemical and ophthalmological measures respectively.

**Table 6:** Pearson’s correlations between the measured parameters.

Parameters	R (Person correlation)	Sig	
Vitamin C (μg/ml) with glutathione (μg/dl)	0.561**	0.001	P^a^
Vitamin C (μg/ml) with macular thickness (µm)—C (OD)	0.365*	0.024	P^a^
GST (U/ml) with macular thickness (µm)—N (out) (OD)	0.352*	0.030	P^a^

*Correlation is significant at the 0.05 level.

**Correlation is significant at the 0.01 level.

^a^Positive correlation.

**Figure 2:**
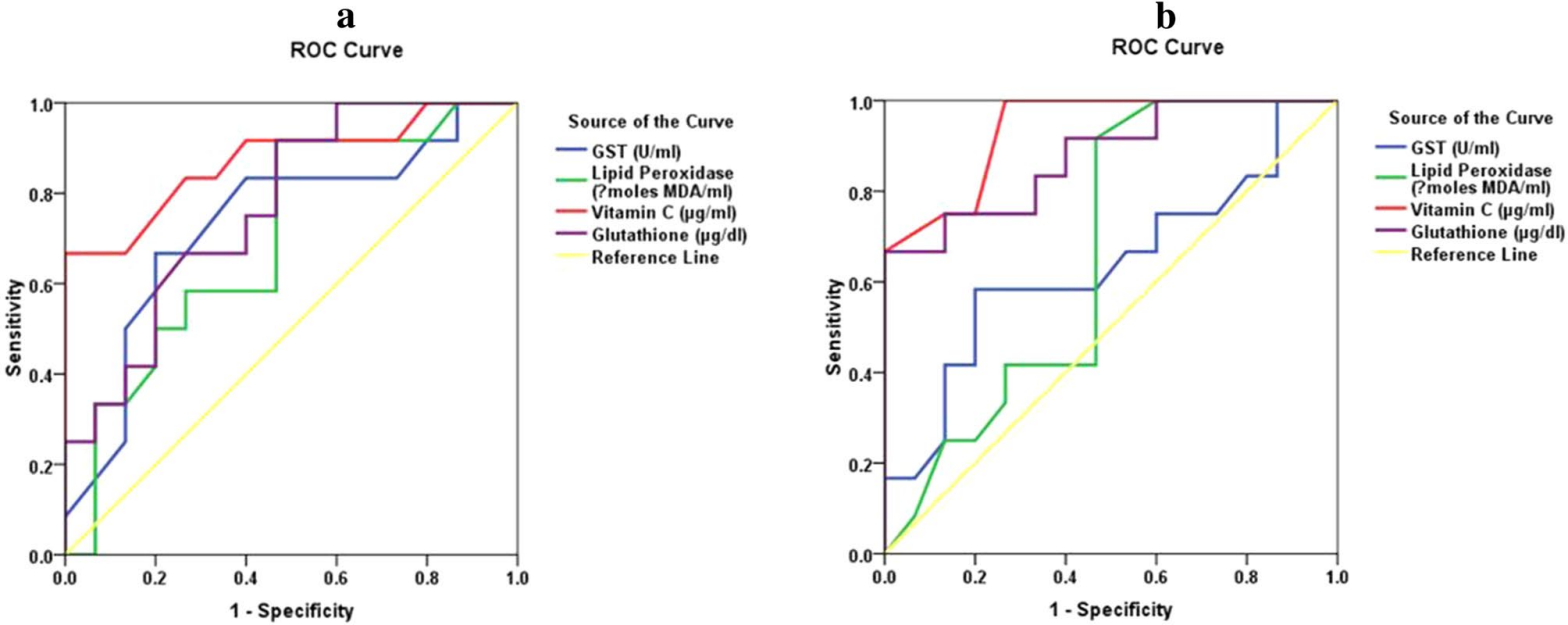
ROC curve of GST (U/ml), lipid peroxidase (μmoles MDA/ml), vitamin C (μg/ml) and glutathione (μg/dl) in **(a)** diabetes without retinopathy; **(b)** diabetes with retinopathy group.

Table 7 and 8 represent the stepwise multiple regression analysis using GST, vitamin C, and GSH as dependent variables. Figure 2a,b demonstrate the ROC analysis of the measured biochemical markers in diabetic with and without retinopathy. Among the four parameters, vitamin C and GSH demonstrate predictive values. Table 9 represents the remarkable increase of the area under the curve (AUC) of combined vitamin C and GSH.

**Table 7:** Multiple regression using stepwise method for GST (U/ml); vitamin C (μg/ml); glutathione (μg/dl) as a dependent variable.

Dependent variable	Predictor variable	Coefficient	P value	Adjusted R square	Model	
					F value	P value
GST (U/ml)	Macular thickness (µm)—N (Out) (OD)	0.027	0.030	0.099	5.075	0.030
Vitamin C (μg/ml)	Glutathione (μg/dl)	0.358	0.001	0.362	21.956	0.001
	Glutathione (μg/dl)	0.374	0.001	0.431	15.019	0.001
	Macular thickness (µm)—T (Out) (OD)	0.084	0.026			
Glutathione (μg/dl)	Vitamin C (μg/ml)	1.058	0.001	0.362	21.956	0.001
	Vitamin C (μg/ml)	1.121	0.001	0.432	15.083	0.001
	Macular thickness (µm)—I (In) (OD)	-0.113	0.025

**Table 8:** ROC-curve of all parameters in all groups.

Parameter	Group	Area under the curve	Cut-off value	Sensitivity %	Specificity %	P value
GST (U/ml)	Group II	0.636	16.450	58.3	80.0	0.232
	Group III	0.733	16.400	66.7	80.0	0.040
Lipid peroxides (μmoles MDA/ml)	Group II	0.661	0.092	91.7	53.3	0.157
	Group III	0.700	0.090	91.7	53.3	0.079
Vitamin C (μg/ml)	Group II	0.936	17.650	100.0	73.3	0.001
	Group III	0.872	22.000	66.7	100.0	0.001
Glutathione (μg/dl)	Group II	0.878	95.955	66.7	100.0	0.001
	Group III	0.769	73.500	91.7	53.3	0.018

**Table 9:** Combined ROC.

Parameter	Group	Area under the curve	Cut-off value	Sensitivity %	Specificity %	P value
Glutathione (μg/dl)	Group II	0.878	95.955	66.7	100.0	0.001
Glutathione (μg/dl) with Macular thickness (µm)—I (In) (OS)		0.967	–	91.7	93.3	0.001
Vitamin C (μg/ml)	Group II	0.872	22.000	66.7	100.0	0.001
Vitamin C (μg/ml) with Macular thickness (µm)—S (In) (OD)		0.933	–	81.8	100.0	0.001

## Discussion

This study proved the effectiveness of metabolic control through medications in ameliorating the effect of oxidative stress as pathophysiological mechanism in many diseases among which is DM. Unexpectedly, higher GSH and vitamin C as anti-oxidants concomitant with non-significantly lower lipid peroxides as marker of oxidative stress were observed in controlled diabetic patients with or without retinopathy compared to healthy non diabetic control.

Ophthalmological results revealed different macular thickness in all quadrants among the three groups with relative increase in group 3 due to DR (Tables 2 and 3).

Retinal nerve fiber layer loss associated with diabetic retinopathy develops before clinically detectable retinal vascular pathology and increases in severity with disease progression^18^ and this is the reason of the usefulness of OCT for the early detection of DR^19^.

The macular thickness differed among groups and increased with disease severity. Previous studies have also reported that macular thickness increased gradually with the progression of DM due to increased retinal vascular permeability and ischemia^20^.

The measured peripapillary RNFLT using 3D-2000 Spectral Domain OCT revealed a similar trend to that of previous studies^21^. The NFL thickness was decreased in clinically detectable DR and the thinning increased with disease severity.

The apoptosis of retinal ganglion cells (RGCs) is intensified in diabetic retinopathy and the death of the RGCs happens early in diabetic eyes^22^. Diabetes-associated NFL loss is caused by ischemia, which is due to retinal microangiopathy^23^. Furthermore, some researchers suggest that neuropathy heralds the vascular changes in early diabetic retinopathy^24^.

Significant changes in lipid metabolism and structure have been reported in diabetic patients suggesting that peroxidative injury may be involved in the development of diabetic complications. Oxidized lipids are able to produce lipid peroxides, also known as malondialdehyde (MDA) as a decomposition product^25^. Our findings of non-significant change in lipid peroxides between diabetic patients with and without DR compared to control (Table 5); are in agreement with the study of Sklodowska et al.^26^ and Pahwa et al.^27^. These studies reported that lipid peroxide concentration was non-significantly different in diabetic patients treated with insulin or oral drug when compared to healthy controls. It is also supported by the recent work of Golizeh et al.^28^, demonstrating non-significant change in lipid peroxides, total antioxidant capacity, serum iron, and ferritin in lifestyle modified and diet-controlled diabetes. On the other hand, it is inconsistent with multiple studies which demonstrate significant elevation of lipid peroxides in diabetic patients compared to controls^29^. This discrepancy can be attributed to the fact that patients examined in the current study or in Sklodowska et al.’s^26^ study were under constant medical care and regular check-ups. This can be explained based on the observations of Sato et al.^30^ who recorded 61% elevation of peroxides in poorly controlled diabetic patients, but unchanged level in controlled diabetic patients. The non-significant increase of lipid peroxides in diabetic patients with retinopathy reported in the present study is not in agreement with the previous study of Aldebasi et. al^31^ which suggested that in Qassim, Saudi patients with proliferative diabetic retinopathy (PDR) demonstrate lipid alterations and elevated lipid peroxides; they recommended glycemic control and lipid lowering drugs to prevent or at least to postpone loss of vision from retinopathy in type 2 diabetics. However, they concluded that, logistic regression shows that lipid peroxides, LDL, and Apo A1 were not associated with PDR, which support our findings.

Based on the same concept on the effectiveness of diabetes control in ameliorating oxidative stress- related markers, the unexpected remarkable increase of GSH in diabetic patients with and without DR compared to control (Table 5), can find support in the work of Sekhar et al.^32^. This study shows that uncontrolled type 2 DM patients have severely deficient synthesis of GSH which can be attributed to limited GSH precursor availability. In contrast, control of diabetes can restore GSH synthesis and lower oxidative stress and oxidant damage even in case of persistent hyperglycemia. Recently, Pahwa et al.^33^ reported non-significant change of GSH in diabetic patients compared to healthy control subjects.

Various toxic molecules formed either from external sources or from the normal metabolism must be detoxified in the body. Glutathione S-Transferase (GST) is an important enzyme catalyzing the detoxification of a variety of toxic molecules through the conjugation with reduced Glutathione (GSH) (the first step in a detoxification pathway)^34^. While many studies reported variable association of GST gene polymorphism and DM, no data related to GST activity is available^35^. The present study shows no significant change in GST activity between DM with and without DR when compared to control (Table 5). This is not in agreement with the recent findings of Pahwa et al.^27^, which showed significant change in the status of GST activity in coronary artery disease (CAD) patients with type 2 DM and insignificant in its activity in CAD without DM type 2. They suggested that GST activity may be induced to combat the increased oxidative stress in case of CAD with DM type 2 and hence can be speculated to have a protective mechanism.

Many enzymatic and non-enzymatic anti-oxidants are present in the body such as vitamin C, superoxide dismutase and glutathione peroxidase which have the ability to control ROS. However, multiple reports have suggested defective vitamin C metabolism concurrent with abnormal leukocytes in diabetic patients^36^. The remarkable increase of vitamin C reported in the present study (Table 5), is further supported by the work of Feillet Coudrey et. al^36^ which demonstrated an increased antioxidant activity in animals with streptozotocin experimentally-induced diabetes. This is further supported by the work of Kojda and Harrison^37^ which shows that controlled or regularly checked up diabetic patients showed increased antioxidants and reduced diabetic complications. Additionally, the decreased rate of retinopathy seen with the use of a combination of vitamin supplements including vitamin C, can explain the non-significant differences in the macular and Optic nerve thickness in diabetic patients with and without retinopathy, reported in the present study (Table 4)^38^. The significant positive correlations between vitamin C, GST, and macular thickness (Table 6 and Fig. 1), together with the contribution of outer and inner macular thickness as predictor variable in the change of GST, vitamin C, and GSH as dependent variables respectively (Table 7) could support the role of controlled diabetes in amending oxidative stress as etiological mechanism of DR. Tables 8 and 9 and Fig. 2 demonstrate the possibility of using vitamin C, GSH, and macular thickness as biomarkers for the early prediction of DR, These three variables recorded high ROC AUCs and satisfactory specificity and sensitivity.

The obtained data can provide more evidence that conventional routine therapy that lowers glycemic levels can reduce the development and progression of oxidative stress in type 2 diabetic patients^39^.

## Conclusion

Hyperglycemia is associated with multiple pathophysiological mechanisms that together promote the aggressiveness of DR. Different approaches should be targeted to treat DR. Among these targets, oxidative stress-related medication can be of great help to stabilize the clinical status that may progress to blindness in uncontrolled type 2 diabetic patients.

### Clinical implication

Oxidative stress has been implicated in the pathogenesis and progression of DR in both type 1 and type 2-diabetes. Identifying the most effective modalities for preventing retinopathy or intervening at an early, asymptomatic stage is necessary to preserve vision. Thus therapeutic correction of oxidant-antioxidant imbalance may be a powerful tool for preventing visual loss associated with DR.

## Materials and method

### Subject recruitment

The prospective observational case control study was carried out between October 2016 and April 2017 at medical retina clinic in King Saud Medical City, Riyadh, Saudi Arabia following the STROBE cross sectional reporting guidelines. The subjects of the study were 39 individuals aged between 40 and 60 years, without a previous history of any endocrine, hepatic, metabolic, cardiovascular and renal disease. Chorioretinitis scars, posterior uveitis, ocular hypertension, glaucoma and previous ocular surgery were also excluded. They were divided into three groups: Group 1 comprised 30 normal eyes of 15 subjects, Group 2 comprised 24 eyes of 12 diabetic patients without retinopathy, and Group 3 comprised 23 eyes of 12 diabetic patients with different grades of retinopathy (8 eyes with maculopathy). Diabetic patients were controlled on their medications (oral hypoglycemic and or insulin, multivitamins and lipid lowering drugs) as they are subjected biannually to regular follow up in our hospital measuring hemoglobin A1c that reflect their glycemic control in last 120 days. Each enrolled subject was given a written informed consent. The study was reviewed and approved by the concerned Ethical Committee. All methods were carried out in accordance with relevant guidelines and regulations of the Declaration of Helsinki.

### Data collection

All Participants underwent a complete ophthalmological examination including visual acuity assessment by Snellen chart, refraction using autorefractometer, slit-lamp examination, Goldmann applanation tonometry, dilated stereoscopic fundus examination using indirect ophthalmoscope for clinical determination of grade of retinopathy, peripapillary retinal nerve fiber layer thickness (RNFLT) and macular thickness measurements using 3D-2000 Spectral Domain optical coherence tomography OCT that uses 360-degree circular scan with 3.4 mm diameter centered on optic disc to measure RNFLT in four quadrants (inferior, superior, nasal and temporal) and line scan for macula measuring inner and outer macular thickness in four quadrants (inferior, superior, nasal, and temporal).

### Blood collection and preparation

Five milliliters of peripheral blood was collected in sterilized plastic vials without anti-coagulate to obtain the serum for biochemical analysis.

#### Biochemical analysis

Lipid oxidation was estimated by the formation of thiobarbituric acid reactive substances (TBARS) as described previously by Ruiz-Larrea et al.^13^. Vitamin C was assayed as previously described by Jagota and Dani^14^. Glutathione was assayed by the method of Beutler et al.^15^ using 5, 5′-dithiobis 2-nitrobenzoic acid (DTNB) with sulfhydryl compounds to produce a relatively stable yellow color. Glutathione S-transferase activity (GST) activity was assessed using an assay kit (Biovision, USA) that was based upon the GST-catalyzed reaction between GSH, GST substrate, and CDNB (1-chloro-2,4-dinitrobenzene)^16^.

### Statistical analysis

SPSS Version 16.0 program was used for the analysis of the obtained data, and results were expressed as mean ± S.D. All statistical comparisons were made using one-way ANOVA test between all groups with multiple comparisons (Dunnett test) to compare each group with the control group in all parameters A significant difference was considered at P value < 0.05. Multiple Regression using Stepwise method was performed for GST, Vitamin C and Glutathione as a dependent variable. The receiver operating characteristics (ROC) curve was used as a fundamental tool to show which biomarkers were predictive in the development of retinopathy. It was performed using the same SPSS Version 16.0 software. Analyzed data were reported in this study. In a ROC curve, the true positive rate (sensitivity) is plotted as a function of the false positive rate (100-specificity) for different cut-off points of a defined parameter. Each point on the ROC curve represents a sensitivity/specificity pair corresponding to a particular decision threshold. The area under the ROC curve is a measure of how well a parameter can be used as biomarker as AUC of 0.5 suggests no discrimination on the basis of ability to diagnose patients with and without the disease; 0.7 to 0.8 is considered acceptable; 0.8 to 0.9 is considered excellent and more than 0.9 is considered outstanding^17^.

### Limitations

Conducting well-designed small studies need careful interpretations. As small studies can provide quick results, it was important to make strong conclusions about oxidative stress as a risk factor for DR, whether the results are positive or not. In addition, data from our studies should be used to design larger confirmatory studies taking into consideration that small studies have their limitations.

### Ethical approval

The protocol of the study was explained to each participant at the time of recruitment and written informed consent was obtained. The research was approved by the research ethical committee of King Saud University. All methods were carried out in accordance with relevant guidelines and regulations of the Declaration of Helsinki.
